# Clinical features of acute fibrinous and organizing pneumonia: An early histologic pattern of various acute inflammatory lung diseases

**DOI:** 10.1371/journal.pone.0249300

**Published:** 2021-04-01

**Authors:** Yasutaka Onishi, Tetsuji Kawamura, Takanori Higashino, Rokuro Mimura, Hiroaki Tsukamoto, Shin Sasaki

**Affiliations:** 1 Department of Respiratory Medicine, National Hospital Organization, Himeji Medical Center, Himeji, Hyogo, Japan; 2 Department of Radiology, National Hospital Organization, Himeji Medical Center, Himeji, Hyogo, Japan; 3 Department of Pathology, National Hospital Organization, Himeji Medical Center, Himeji, Hyogo, Japan; Medizinische Hochschule Hannover, GERMANY

## Abstract

**Background:**

Acute fibrinous and organizing pneumonia (AFOP) is a rare histologic pattern of acute lung involvement with intra-alveolar fibrin deposition. However, the clinical significance of the pathological findings of AFOP remains unclear. This study aimed to explore the clinical significance of AFOP through a comprehensive clinical examination.

**Methods:**

The medical records of patients with lung diseases accompanied by the pathological finding of intra-alveolar organization between January 2010 and December 2019 were retrospectively reviewed. The clinical and radiological findings were compared between the groups with and without the histologic pattern of AFOP.

**Results:**

We identified 34 patients with AFOP (AFOP group) and 143 without AFOP (non-AFOP group). The underlying diseases of the AFOP group were as follows: 19 patients had cryptogenic organizing pneumonia (OP), 5 had connective tissue diseases, 3 had radiation pneumonitis, 3 had chronic eosinophilic pneumonia, 2 had myelodysplastic syndromes, and 2 had drug-induced pneumonia. Fever was more common, the time from symptom onset to biopsy was shorter, and the serum C-reactive protein level was higher in the AFOP group than in the non-AFOP group. On high-resolution computed tomography, 85% of patients had OP pattern, and halo sign was more common in the AFOP group. Corticosteroids were effective in 94% of the patients in the AFOP group; however, recurrences were more frequent, and a higher corticosteroid dose was needed during recurrence.

**Conclusions:**

AFOP might be an early phase of a histologic pattern associated with known etiologies. In addition, it could be a marker indicating intense inflammatory diseases with a tendency of recurrence.

## Introduction

In 2002, Beasley et al. reported 17 cases of lung injury with a unique histologic pattern characterized by intra-alveolar fibrin deposition, and the pattern was proposed to represent acute fibrinous and organizing pneumonia (AFOP) [1]. In addition, the study suggested AFOP as a novel clinical entity because its clinical course might be distinct from existing entities. The number of case reports has increased since AFOP was described as a rare histologic pattern in the American Thoracic Society/European Respiratory Society statement on idiopathic interstitial pneumonias in 2013 [2]. There are various ways of interpreting AFOP, whether it is one of the distinct clinical entities or simply a universal tissue reaction of known etiologies, such as collagen vascular disease, hematologic diseases, or idiopathic interstitial pneumonias (like cryptogenic organizing pneumonia [COP]) [1–7]. Previous studies have shown that COP patients with intra-alveolar fibrin deposition tend to be associated with a high recurrence rate compared to those without intra-alveolar fibrin deposition [8–10]. However, few reports have described the clinical differences between patients with and without pathological AFOP and its etiology, and the clinical significance of the presence of AFOP remains unclear. Moreover, since the original AFOP report has been published in 2002, additional important autoantibodies implicated in the etiology of lung injury, such as myositis-specific autoantibodies, have been identified and can now be examined [11, 12].

This study aimed to explore the clinical significance of AFOP through a comprehensive clinical and radiological examination.

## Material and methods

### Study population

A total of 296 consecutive patients examined at the National Hospital Organization Himeji Medical Center from January 2010 to December 2019 and with a histological finding of organizing pneumonia (OP) on forceps transbronchial lung biopsy (TBLB) or surgical lung biopsy (SLB) were retrospectively evaluated. After multidisciplinary discussion on disease behavior according to a previous report [1], patients with the following diagnoses were excluded: infectious disease (bacterial pneumonia, lung abscess, and pyothorax), lung cancer-associated OP, inflammatory nodule, and hypersensitivity pneumonitis. Patients with a chronic course (> 2 months from the onset), asymptomatic cases, focal OP, which is difficult to distinguish from bacterial pneumonia [13], and patients without chest high-resolution computed tomography (HRCT) scan were also excluded.

The study was conducted in accordance with the amended Declaration of Helsinki. The study design was approved by the institutional review board of the National Hospital Organization Himeji Medical Center (IRB No. 2019–41). Informed consent was obtained from all patients using an opt-out approach; the research proposal was documented in patient information leaflets and posted on the website of our hospital in accordance with the ethical guidelines for medical research involving human subjects in Japan. This procedure was approved by the ethics committee. All data were fully anonymized before being accessed, and the period for which patient information was accessed was between December 2019 and January 2021.

### Radiological assessment

All patients were required to undergo a chest HRCT scan within 1 month of their first visit. HRCT was performed using a 16- or 80-detector row CT scanner (Aquilion; Canon Medical Systems, Tokyo, Japan). The slice thickness was 1–2 mm, with slices obtained at 1–5 mm intervals. Blinded to all clinical information, each HRCT scan was evaluated independently by two specialists: an expert chest radiologist (T.H.) with 20 years of CT-scanning experience for interstitial lung diseases and a pulmonary specialist (T.K.) with 25 years of clinical experience with interstitial lung diseases.

**Fig 1:**
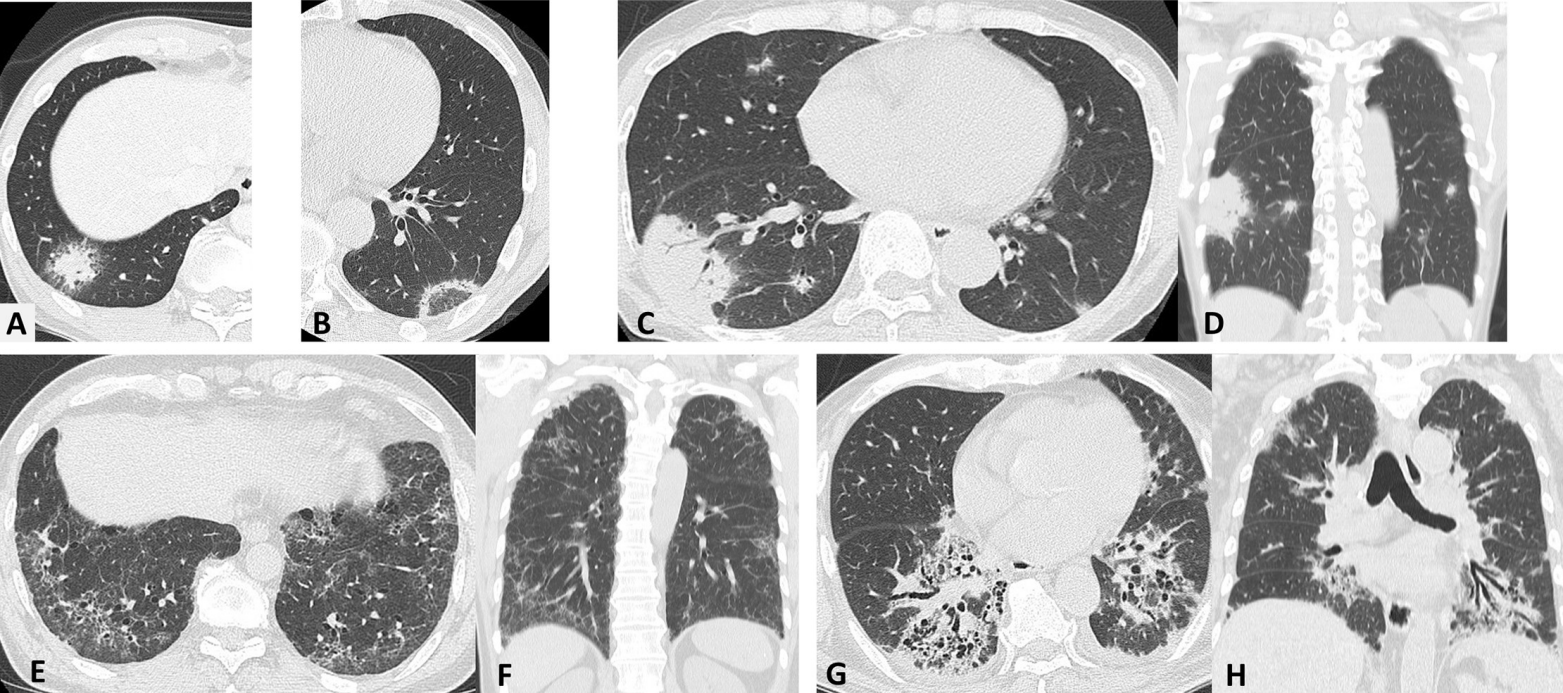
HRCT images of representative signs and CT patterns. (A) Halo sign: the nodular consolidation surrounded by GGO. (B) Reversed halo sign: rounded area of GGO surrounded by a ring of consolidation. (C, D) OP pattern: axial and coronal HRCT images showing patchy, peripheral predominant multiple consolidation with air bronchogram. (E, F) NSIP pattern: diffuse, subpleural predominant reticulation, and GGO with traction bronchiectasis. (G, H) NSIP and OP pattern: bilateral lower zone, peribronchovascular predominant consolidation, and GGO with traction bronchiectasis. HRCT, high-resolution computed tomography; CT, computed tomography; GGO, ground-glass opacity; OP, organizing pneumonia; NSIP, nonspecific interstitial pneumonia.

The checkpoints assessed during image reading were as follows: the presence of consolidation, ground-glass opacity (GGO), air-bronchogram, halo sign, and reversed halo sign. The halo sign (Fig 1A) referred to a less dense or ground-glass area of lung attenuation (compared with the central nodule or mass) that extends around the entire circumference of a central nodule or mass [14]. The reversed halo sign (Fig 1B) referred to central GGO surrounded by a denser consolidation of a crescentic or ring shape of at least 2 mm in thickness [14]. The number of affected lobes was also evaluated. The distribution of abnormalities was classified into upper (right or left upper lobe), middle (right middle lobe or lingula), and lower (right or left lower lobes) predominance.

In addition, the HRCT pattern was categorized into four groups: OP pattern, nonspecific interstitial pneumonia (NSIP) pattern, NSIP with OP pattern, and other pattern. OP pattern (Fig 1C and 1D) was defined as patchy, peripheral, multiple airspace consolidations, with a certain degree of GGO, and often associated with air bronchogram [2, 13]. NSIP pattern (Fig 1E and 1F) referred to diffuse, subpleural, bilateral lower zone predominance associated with reticulation, GGO, traction bronchiectasis, and volume loss, and often homogeneous and peribronchovascular distribution [2, 15]. NSIP with OP pattern (Fig 1G and 1H) referred to bilateral lower zone predominance associated with GGO, airspace consolidation along the bronchovascular bundle, a certain degree of volume loss, and traction bronchiectasis, thus combining some traits of the NSIP and OP patterns [16].

### Histological assessment

All specimens were reviewed by a pathologist (R.M.) with 20 years of diagnostic experience with interstitial lung diseases. First, the presence of organizing intra-alveolar fibrin was screened on hematoxylin and eosin (H&E) staining. When intra-alveolar fibrin was detected using H&E staining, additional phosphotungstic acid-hematoxylin staining was performed to assess fibrin deposition [10]. The histologic pattern was then considered as AFOP when the pathological findings met the criteria proposed by Beasley et al. [1], with three major features including (1) dominant finding of organizing intra-alveolar fibrin, (2) OP, and (3) patchy distribution without negative findings, including hyaline membranes, conspicuous eosinophils, bronchopneumonia, abscess formation, and granulomatous inflammation.

### Clinical assessment

The following clinical and radiological characteristics were compared between patients with AFOP (AFOP group) and without AFOP (non-AFOP group): clinical symptoms (fever, cough, dyspnea), oxygen saturation, time between the symptom presentation and biopsy, laboratory data including C-reactive protein (CRP), serum Krebs von den Lungen-6 (KL-6), and surfactant protein-D levels. Antinuclear antibody and disease-specific autoantibodies, including myositis-specific antibodies, were examined depending on the clinical conditions. Myositis-specific antibodies were detected using the MESACUP™ anti-aminoacyl tRNA synthetase (ARS) test (Medical & Biological Laboratories Co., Ltd.), which is based on enzyme-linked immunosorbent assays (ELISAs) [11] (anti-Jo-1, anti-PL-7, anti-PL-12, anti-EJ, and anti-KS antibodies) and/or Euroline Myositis Profile 3 kits (EUROIMMUN) [17] (anti-Mi-2, anti-Ku, anti-PM-Scl100, anti-PM-Scl75, anti-SRP, anti-Jo-1, anti-PL-7, anti-PL-12, anti-OJ, anti-EJ, and anti-Ro-52 antibodies) based on line blot immunoassays and/or anti-melanoma differentiation-associated gene (MDA) 5 antibody based on ELISAs (Medical & Biological Laboratories Co., Ltd.) [12]. Bronchoalveolar lavage fluid findings, pulmonary function tests, HRCT findings, the initial dose of corticosteroids, treatment response, initiation of immunosuppressants, follow-up period, recurrence rate, corticosteroid dose at the recurrence, number of recurrences, and prognosis were also examined. The definition of a good response for initial treatment was an improvement in symptoms and radiological findings that allowed discharge.

Regarding the steroid tapering regimen, the initial dose of oral corticosteroids was 0.5–0.8 mg/kg/day depending on the clinical manifestation (the patient’s symptoms, extent of the shadow, clinical course, or the underlying disease) [9]. For patients who met the criteria for acute lung injury (PaO_2_/FiO_2_ < 300 mmHg), the initial dose was 1.0 mg/kg/day after 3 days of intravenous methylprednisolone pulse (1000 mg/day). The treatment was basically tapered to 5–10 mg/day every 1–2 weeks depending on the clinical course. For patients who had relapsed, corticosteroids were re-administered or increased to about half of the initial dose and tapered in the same manner monitoring the treatment effects. In addition, administration of immunosuppressants or erythromycin was considered based on the underlying disease and the frequency of recurrences.

### Statistical analyses

Continuous variables were expressed as mean ± standard deviation or median [25^th^–75^th^ quartiles]. The Mann–Whitney *U* test was used for nonnormally distributed variables, and Student’s *t*-test was used for analyzing normally distributed variables. Categorical variables were compared with the use of either the chi-square test or Fisher’s exact test. A *P*-value of < 0.05 was considered statistically significant. IBM SPSS Statistics 23 (IBM Corp., Armonk, NY, USA) was used for all statistical analyses.

## Results

### Baseline clinical and serological characteristics

**Fig 2:**
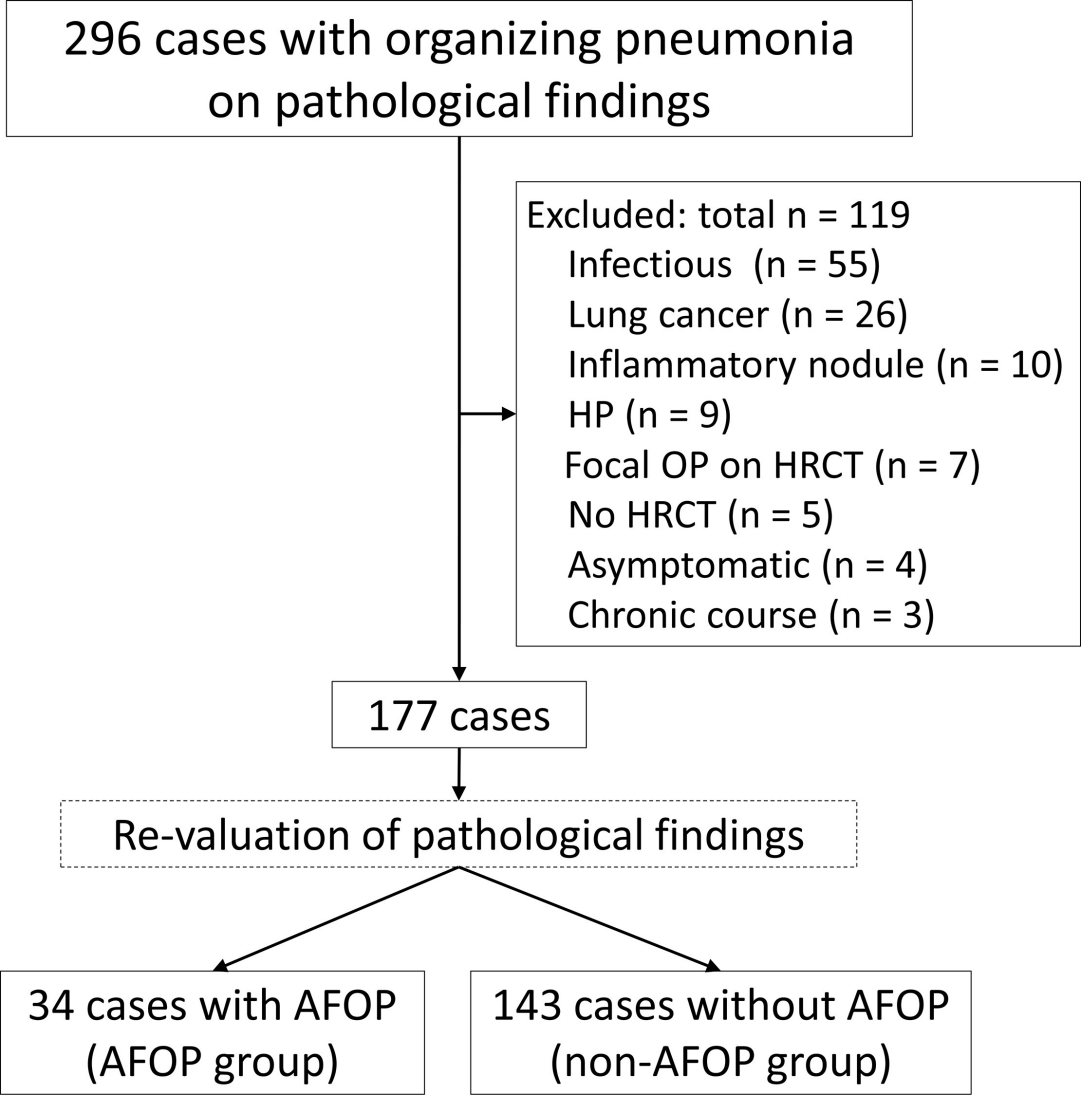
Flowchart of patient disposition. Final diagnosis in 34 patients with AFOP (AFOP group) and 143 patients without AFOP (non-AFOP group). AFOP, acute fibrinous and organizing pneumonia; HP, hypersensitivity pneumonia; OP, organizing pneumonia.

The flowchart of patient selection is shown in Fig 2. Of the 296 patients, 119 were excluded, and the remaining 177 patients were enrolled in this study. Of these, 34 patients who met the pathological criteria for AFOP were classified into the AFOP group, and the remaining 143 patients were classified into the non-AFOP group. Clinical and radiological characteristics were compared between the two groups (Table 1).

**Table 1:** Underlying diseases of patients.

	AFOP group	non-AFOP group
	n = 34	n = 143
COP	19 (56)	61 (43)
Autoimmune disease	4 (12)	42 (29)
ANCA-associated vasculitis	2 (6)	5 (3)
Rheumatoid arthritis	1 (3)	23 (16)
PM/DM	1 (3)	13 (9)
SSc	0	1 (1)
CEP	3 (9)	18 (13)
Radiation pneumonitis	3 (9)	3 (2)
Drug-induced pneumonia	2 (6)	5 (3)
Hematologic disease	2 (6)	3 (2)
IgG4-related disease	1 (3)	1 (1)
Idiopathic NSIP	0	10 (7)

Data are presented as numbers (%). AFOP, acute fibrinous and organizing pneumonia; COP, cryptogenic organizing pneumonia; ANCA, anti-neutrophil cytoplasmic antigen; PM/DM, polymyositis/dermatomyositis; SSc, systemic sclerosis; CEP, chronic eosinophilic pneumonia; NSIP, nonspecific interstitial pneumonia.

**Fig 3:**
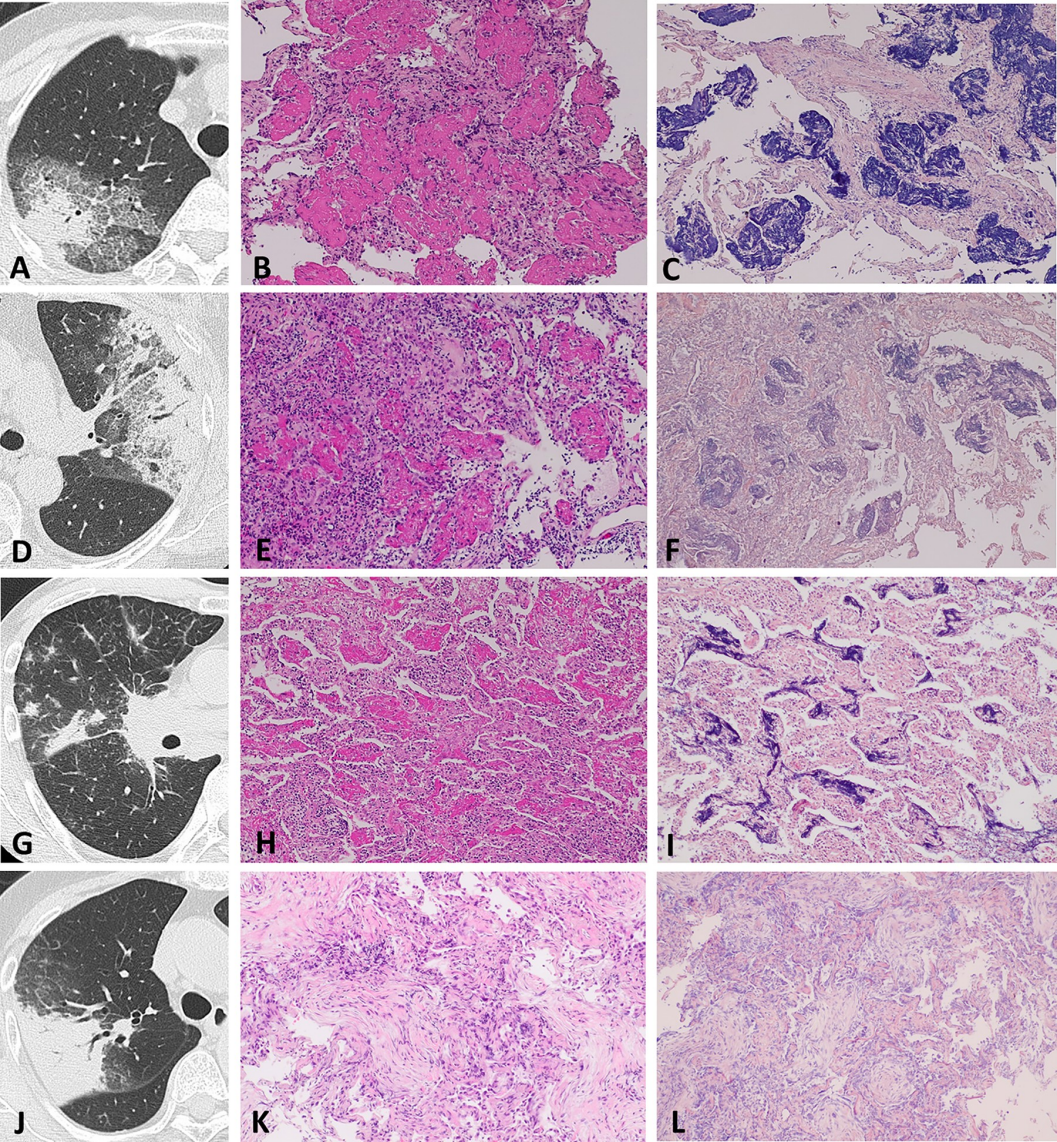
Representative radiological and histological findings of AFOP and non-AFOP. (A–C) Patient 1, COP with AFOP. (A) The HRCT shows subpleural patchy airspace consolidation with GGO in the right upper lobe. (B, C) Histological findings obtained by TBLB show organizing pneumonia and multiple foci of fibrin in the alveoli, which are consistent findings of AFOP. (D–F) Patient 2, radiation pneumonitis with AFOP. (D) Subpleural airspace consolidation with GGO in the left upper lobe. (E, F) Histological findings obtained by TBLB showing multiple foci of intra-alveolar fibrin and infiltration of lymphocytes in the alveolar wall. (G–I) Patient 3, IgG4-related pulmonary disease with AFOP. (G) Subpleural, irregular form of consolidation and thickened intra-alveolar septa. (H, I) Histological findings obtained by surgical lung biopsy show intra-alveolar fibrin and infiltration of plasmacytes and lymphocytes in the alveolar wall. (J–L) Patient 4, COP without AFOP. (J) Subpleural airspace consolidation in the right upper lobe. (K, L) Histological findings obtained by TBLB show organizing pneumonia with intra-alveolar granulation. No intra-alveolar fibrin deposition can be observed. (B, E, H, K) Hematoxylin and eosin staining (×10). (C, F, I, L) Phosphotungstic acid-hematoxylin staining (×10). AFOP, acute fibrinous and organizing pneumonia; COP, cryptogenic organizing pneumonia; HRCT, high-resolution computed tomography; GGO, ground-glass opacity; TBLB, transbronchial lung biopsy.

Biopsy specimens were obtained by TBLB in 160 patients (AFOP group: 32, non-AFOP group: 128) and SLB in 17 patients (AFOP group: 2, non-AFOP group: 15). Representative radiological and histological findings of three cases with AFOP and a COP case without AFOP are presented in Fig 3.

The underlying diseases of patients in the AFOP group were as follows (Table 1): 19 had COP, 4 had autoimmune diseases (2 anti-neutrophil cytoplasmic antigen [ANCA]-associated vasculitis, 1 rheumatoid arthritis, and 1 anti-MDA5 antibody-positive polymyositis/dermatomyositis [PM/DM]), 3 had chronic eosinophilic pneumonia (CEP), 3 had radiation pneumonitis, 2 had drug-induced pneumonia (afatinib and pembrolizumab), 2 had myelodysplastic syndrome (MDS), and 1 had IgG4-related disease. For the non-AFOP group, 61 had COP, 42 had autoimmune diseases (23 rheumatoid arthritis, 13 PM/DM, 5 ANCA-associated vasculitis, 1 systemic sclerosis), 18 had CEP, 5 had drug-induced pneumonia, 3 had radiation pneumonitis, 3 had hematologic diseases (1 MDS, 1 adult T-cell leukemia, and 1 autoimmune hemolytic anemia), 10 had idiopathic NSIP, and 1 had IgG4-related disease.

The clinical characteristics of both groups are presented in Table 2. There was no difference in the mean age, proportion of men, smoking history, and oxygen saturation on their first visit between the groups. Regarding clinical symptoms, fever was more common in the AFOP group (AFOP group vs. non-AFOP group: 74% vs. 45%, *P* = 0.006), and the period from symptom onset to biopsy was significantly shorter (16.1 ± 7.5 days vs. 25.6 ± 11.2 days, *P* < 0.001) in the AFOP group than that in the non-AFOP group.

**Table 2:** Clinical characteristics of patients.

	AFOP group	non-AFOP group	*P*-value
	n = 34	n = 143	
Age, years	66.4 ± 13.2	66.3 ± 12.9	1
Male	19 (56)	72 (50)	0.6
Never smoker	17 (50)	71 (50)	1
Clinical symptoms			
Fever	25 (74)	65 (45)	0.006
Cough	25 (74)	98 (69)	0.7
Dyspnea	9 (26)	37 (26)	1
Oxygen saturation, %	94.8 ± 2.2	95.1 ± 2.4	0.4
Days from onset to biopsy	16.1 ± 7.5	25.6 ± 11.2	< 0.001
Laboratory data			
WBC, ×10^3^/μL	9.3 ± 2.2	8.6 ± 2.7	0.2
CRP, mg/dL	12.8 [7.4–18.1]	3.7 [1.1–7.7]	< 0.001
KL–6, U/mL	285 [208–423]	432 [268–967]	< 0.001
SP–D, ng/mL	112 [74–210]	116 [71–214]	0.9
ANA positive	2 (6)	16 (11)	0.5
Myositis autoantibodies positive*	1 (5)	11 (12)	0.7
BALF^†^			
Eosinophils, %	7 [1.0–14.0]	8 [3.0–18.0]	0.2
Neutrophils, %	9.5 [5.8–16.8]	8.0 [4.0–14.0]	0.2
Lymphocytes, %	59.8 [30.9–75.2]	46.0 [26.8–65.0]	0.2
Macrophages, %	9.5 [5.8–24.3]	19.5 [8.0–42.0]	0.06
Pulmonary function test^‡^			
%FVC	92.2 ± 10.1	87.9 ± 16.9	0.3
%DLco	84.2 ± 28.6	81.0 ± 19.6	0.5

Categorical variables are expressed as numbers (%), and continuous variables are expressed as mean ± standard deviation or median [25^th^–75^th^ quartiles].

AFOP, acute fibrinous and organizing pneumonia; ANA, antinuclear antibody; BALF, bronchoalveolar lavage fluid; CRP, C-reactive protein; DLco, diffusing capacity for carbon monoxide; FVC, forced vital capacity; KL-6, Krebs von den Lungen-6; SP-D, surfactant protein-D; WBC, white blood cells.

*Data were available for 20 and 93 patients in each group. ^†^Data were available for 29 and 118 patients in each group. ^‡^Data were available for 20 and 111 patients in each group.

Regarding the laboratory data, serum CRP levels were higher and serum KL-6 levels were lower in the AFOP group than those in the non-AFOP group. For myositis-specific autoantibodies, there was 1 positive case (anti-MDA5 antibody) in the AFOP group and 11 positive cases in the non-AFOP group (EJ: 2 cases, Jo-1: 2, MDA5: 2, PL-7: 1, PM-Scl75: 1, OJ: 1, SRP: 1, and Mi-2: 1). There were no differences in the other laboratory data, bronchoalveolar lavage fluid findings, and pulmonary function tests between the two groups.

### Radiological findings

Patients in the AFOP group were more likely to have the halo sign (21% vs. 6%, *P* = 0.01) than those in the non-AFOP group, whereas no difference was found in the presence of consolidation, GGO, air-bronchogram, the reversed halo sign, and the numbers of affected lobes and lung zones between the two groups (Table 3).

**Table 3:** HRCT findings.

	AFOP group	Non-AFOP group	*P*-value
	n = 34	n = 143	
Consolidation	34 (100)	132 (92)	0.1
Ground-glass opacity	34 (100)	141 (99)	1
Air-bronchogram	28 (82)	113 (79)	0.8
Halo sign	7 (21)	8 (6)	0.01
Reversed halo sign	1 (3)	15 (10)	0.3
Number of affected lobes	3.8 ± 1.2	3.9 ± 1.1	0.3
Lung predominance			
Upper	33 (97)	131 (92)	0.5
Middle	26 (76)	110 (77)	1
Lower	32 (94)	140 (98)	0.3
CT patterns			
OP pattern	29 (85)	104 (73)	0.2
NSIP pattern	0	15 (11)	0.08
NSIP with OP pattern	1 (3)	19 (13)	0.1
Other pattern	4 (12)	5 (3)	0.07

Data are presented as numbers (%) or mean ± standard deviation. HRCT, high-resolution computed tomography; AFOP, acute fibrinous and organizing pneumonia; OP, organizing pneumonia; NSIP, nonspecific interstitial pneumonia; CT, computed tomography.

Similarly, there was no difference between the two groups regarding their CT patterns. In the AFOP group, the OP pattern accounted for 85% (29/34) of cases followed by other pattern which accounted for 12% of cases (4 cases: ANCA-related vasculitis, IgG4-related disease, drug-induced pneumonia [afatinib], and MDS), and NSIP with OP pattern which accounted for 3% of cases (1 case of anti-MDA5 antibody-positive PM/DM). None of the AFOP patients showed an NSIP pattern.

### Therapeutic course

**Fig 4:**
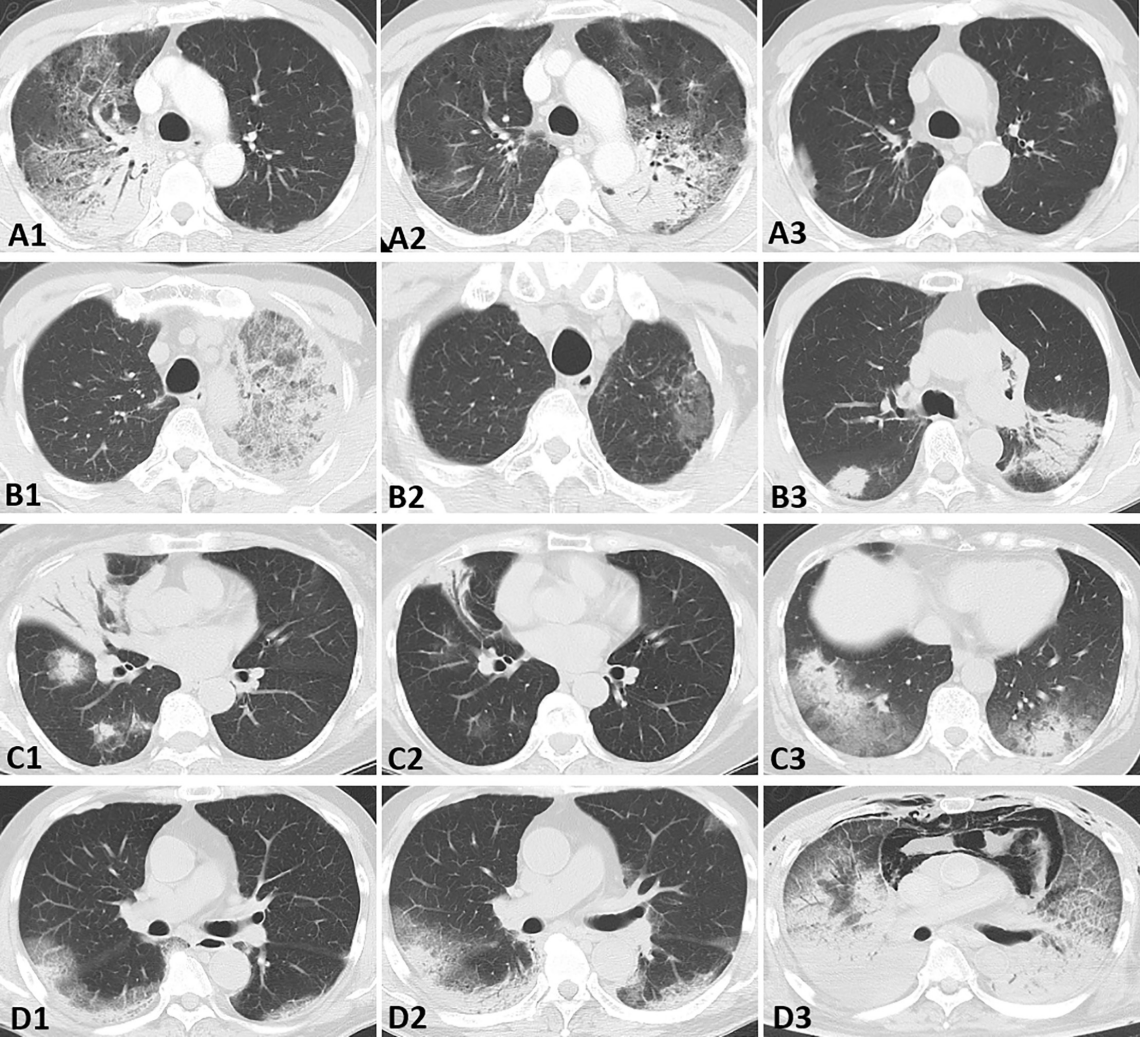
Representative clinicoradiological courses of four patients in the AFOP group. (A1-3) Patient 1. Chest CT images in a 63-year-old man with COP. (A1) Consolidation and GGO with air bronchogram in the right upper lobe at diagnosis. (A2) Three months after the administration of corticosteroids, relapse was observed in the left upper lobe. (A3) One year after the diagnosis, most of the shadow had improved, but the patient continued to experience relapses. (B1-3) Patient 2. A 65-year-old man with advanced lung cancer treated with anti-PD-1 antibody. (B1) Drug-induced pneumonia with consolidation and GGO in the left upper lobe that had developed four months after the initiation of the anti-PD-1 antibody therapy. (B2) Three weeks after corticosteroid treatment. (B3) One year after the diagnosis of drug-induced pneumonia, relapse was observed in both lower lobes. (C1-3) Patient 3. A 67-year-old woman after radiation therapy for right breast cancer. (C1) Radiation pneumonitis with halo sign developed six months after the radiation therapy. (C2) Three months after the treatment with corticosteroids. (C3) Six months after the diagnosis of radiation pneumonitis, relapse of radiation pneumonitis was observed. The therapeutic course was good with increased corticosteroid dosage. (D1-3) Patient 4. A 74-year-old man with anti-MDA5 antibody-positive dermatomyositis and rapidly progressive interstitial lung disease. (D1) Consolidation and GGO along the peripheral bronchovascular bundle predominantly in the lower lobes at the initial visit. (D2) At the time of admission, the shadow had progressed further within a week. (D3) Despite intense treatment with biweekly intravenous cyclophosphamide pulse (1000 mg/day), tacrolimus (5 mg/day), plasma exchange, and methylprednisolone pulses (1000 mg/day) followed by oral corticosteroid (1 mg/kg/day), extensive infiltrative shadow and pneumomediastinum had developed. The patient died due to acute respiratory failure four weeks after the initial visit.

Treatment-related characteristics are presented in Table 4. Corticosteroids were administered in 32 of the 34 patients (94%) in the AFOP group and 122 of the 143 patients (85%) in the non-AFOP group, and most of them showed a good response (94% and 98%, respectively). A methylprednisolone pulse was administered in 4 patients in both groups, and immunosuppressants were added for initial therapy in 3 patients (9%) of the AFOP group (intravenous cyclophosphamide [IVCY] + tacrolimus: 1 case, IVCY + azathioprine: 1 case, IVCY: 1 case) and in 24 patients (17%) of the non-AFOP group (cyclosporine: 10 cases, azathioprine: 6 cases, IVCY + cyclosporine: 4 cases, tacrolimus: 3 cases, IVCY: 1 case). Despite intensive treatment, 2 cases in the AFOP group (radiation pneumonitis and anti-MDA5 antibody-positive PM/DM) and 4 cases in the non-AFOP group (anti-PM-Scl75 antibody-positive PM/DM, RA-related NSIP, and 2 cases of ANCA-related vasculitis) died because of respiratory failure. Representative clinicoradiological courses of patients in the AFOP group are presented in Fig 4. The initial dose of steroid administered, the recurrence rate, and the steroid dose administered for recurrences were higher, the number of days from onset to recurrence was lower, and increased recurrence rates were observed in the AFOP group compared to the non-AFOP group. There was no difference in the rate of use of immunosuppressants, the response rate of initial treatment, and the follow-up period between the two groups.

**Table 4:** Treatment-related characteristics of patients.

	AFOP group	non-AFOP group	*P-*value
	n = 34	n = 143	
Steroid treatments	32 (94)	122 (85)	0.3
Initial treatment response	30 (94)	120 (98)	0.1
Immunosuppressant treatments	3 (9)	24 (17)	0.8
Initial steroid dose (mg/kg/day)	0.6 [0.6–0.9]	0.6 [0.5–0.8]	0.02
Follow-up period (days)	712 [390–459]	869 [373–1748]	0.3
Relapsed cases	26 (76)	51 (36)	< 0.001
Days from onset to recurrence (days)	163 [102–255]	329 [173–757]	0.001
Steroid dose at recurrence (mg/day)	5 [2.5–9.4]	3 [1.0–5.0]	0.04
Recurrence during follow-up (times)	1.8 ± 1.7	0.6 ± 1.0	< 0.001
Death from respiratory failure	2 (6)	4 (3)	0.3

Categorical variables are expressed as numbers (%), and continuous variables are expressed as mean ± standard deviation or median [25^th^–75^th^ quartiles]. AFOP, acute fibrinous and organizing pneumonia.

Regarding the therapeutic course after relapse, most of the AFOP patients responded well to the re-administration or increase in corticosteroids dose except for two patients who died of respiratory failure. Erythromycin was administered to two patients and azathioprine to one patient with recurrent relapses, all of whom experienced subsequent relapses.

## Discussion

More than 100 cases of AFOP have been reported to date and have contributed to a better understanding of their clinical characteristics; however, the clinical significance of pathological AFOP remains unclear. Some studies have reported that AFOP might simply represent a universal histologic pattern associated with various respiratory diseases and that their clinical course depends on the severity of the underlying condition [2, 5, 8]. Moreover, a large number of idiopathic cases have been associated with poor prognosis, which was reported in the original study; however, other studies have reported a good response to steroid therapy and good prognosis [1, 5, 18].

Although AFOP is thought to be a rare histologic pattern, it was not rare in our study (19%, 34/177 cases). The most frequent underlying disease in the AFOP group was COP (56%, 19/34 cases) with no clinical and serological evidence of other background disorders. This category was followed by 4 cases that met the criteria of autoimmune disease [19–21]. Myositis-related interstitial pneumonia was assumed to be more frequent [4]; conversely, AFOP was not detected in any of the 13 cases of PM/DM (including 5 cases confirmed by surgical lung biopsy). However, it is important to note that approximately 90% of the biopsy samples were obtained by TBLB, which may have led to an underestimation of AFOP pathology.

**Fig 5:**
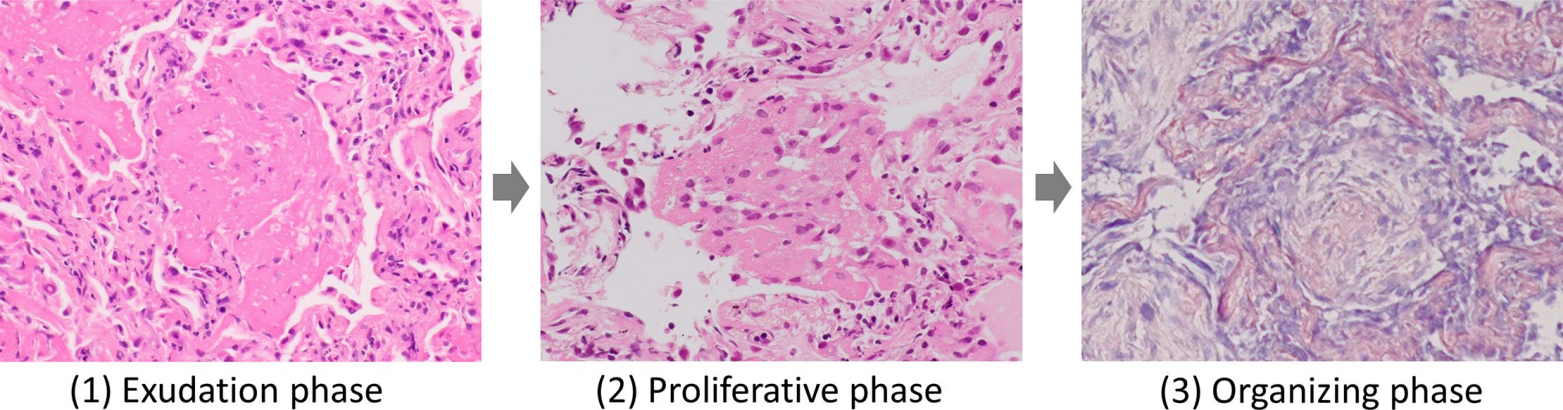
Healing process after lung injury. (1) Exudation phase: inflammation triggers exudation of fibrinogen into the alveolar space, and fibrinogen is converted into fibrin matrix (hematoxylin and eosin staining, ×20). (2) Proliferative phase: fibroblasts and macrophages migrate into the fibrin matrix (hematoxylin and eosin staining, ×20). (3) Organizing phase: the fibrin matrix is replaced by collagen tissue (phosphotungstic acid-hematoxylin staining, ×20).

The analysis of our results with a short period from symptom onset to biopsy and high serum CRP levels in the AFOP group suggests that the following two conditions may be necessary for the histological diagnosis of AFOP. The first condition is the timing of the lung biopsy. Wound healing after lung injury is a staged process as follows (Fig 5) [22]: (1) in the exudation phase, inflammation leads to increased vascular permeability and influx of plasma proteins, including fibrinogen, and fibrinogen is converted into a fibrin matrix by activated procoagulants [23]; (2) in the proliferative phase, fibroblasts and macrophages migrate into, adhere to, and proliferate in fibrin matrices in the presence of exudated plasma fibronectin, which is known as cell adhesion factor [24, 25]; (3) in the organizing phase, fibrin clots are replaced by collagen with a reaction of plasmin and are removed by endocytosis followed by degradation in lysosomes in the inflammatory cells [26]. In addition, an intraluminal Masson body, which is a common pathological finding in COP, is considered to be the organization of fibrinous exudates [27]. This means that the existence of a Masson body indicates past intra-alveolar fibrin. From the above, it is assumed that most inflammatory lung diseases have a period of intra-alveolar fibrin development. Thus, intra-alveolar fibrin, a core finding of AFOP, is more likely to be observed when lung biopsy is conducted early after symptom onset.

The second condition is an intense inflammatory status resulting from an underlying disease; prominent symptoms such as high fever urge patients to visit hospitals, and patients with high serum CRP levels are encouraged to have an earlier medical examination. Moreover, intense inflammation enhances vascular permeability of the alveolar-capillary membrane resulting in more extensive deposition of fibrin in the alveolar space. In addition, a previous report has also shown that high CRP levels were associated with intra-alveolar fibrin deposition [28]. Therefore, the histologic pattern of AFOP might be observed more frequently when these two conditions are met: biopsy in the early phase and intense inflammatory status. When disease progression exceeds tissue repair, extensive deposition of extravascular fibrin acts as a powerful inflammatory mediator and causes excessive inflammation and dysregulation of the coagulation system, which could lead to life-threatening outcomes [26, 29–31]. In such cases, immediate intervention including immunosuppressants is required for eliminating excess fibrin and for minimizing lung injury.

The frequency of the halo sign in the AFOP group in our study suggests that the time course between disease onset and CT imaging might be important. The halo sign is commonly present in infectious diseases as typified by invasive pulmonary aspergillosis, and it is also associated with the acute phase of inflammatory lung diseases, such as CEP and COP [32–34]. The halo sign was observed in 8.5% (15/177 cases) of all cases in our study, which was comparable to the proportion (8.3%) reported in a previous study [34]. In addition, Inoue et al. proposed a time course of CT imaging of COP illustrating the initial finding of nodular opacity that enlarged concentrically, followed by resolution in the central lesion of the consolidation as observed in the reversed halo sign [35]. Thus, the halo sign might be an initial radiological manifestation of lung disease with AFOP as with COP.

The serum KL-6 level is also influenced by the time course of lung injury, and serum KL-6, produced mainly by “regenerating” type II pneumocytes, is a useful biomarker for detecting interstitial pneumonia [36]. Therefore, the peak level of serum KL-6 can be observed late after the onset of the disease [37], and thus, the serum level might be lower in the AFOP group.

For the treatment course, both groups showed a favorable response to corticosteroids; however, more recurrences were observed in the AFOP group, and 6% (2/34 cases) of the patients were unresponsive despite intensive therapy. In the original report by Beasley et al., 7 of the 17 patients with AFOP received corticosteroids; however, 5 cases resulted in a fulminant course [1]. Given that the response to treatment depends on the severity of the underlying disease, in the case of patients who do not respond to steroid treatment, underlying diseases such as hematologic or autoimmune diseases should be pursued as much as possible [6, 38–40]. The rate of recurrence appears to be relatively high for AFOP. Nishino et al. showed a recurrence rate of 60% (6/10 cases) in COP patients with AFOP [8], and the result was similar to that of our study with a 76% recurrence rate. According to these results, the presence of AFOP may have clinical significance for recurrence after steroid treatment.

There are several limitations to this study. Although our study included the largest cohort of patients with AFOP to date, patients were from a single municipal hospital, and the number of cases was limited; the study was also a retrospective analysis. Since this study was conducted on Asian people, it is not clear whether the results apply to Caucasians and other races. In addition, most biopsy specimens were obtained by TBLB, which provides relatively smaller specimens compared to SLB. The transbronchial approach is recommended before proposing SLB because TBLB can yield diagnostic material in the majority of patients with the pathological finding of OP [5, 7, 13, 41, 42]; however, our study might underestimate the number of patients with NSIP pattern or NSIP with OP pattern because their lung involvement was not fully investigated pathologically. Moreover, considering the value of oxygen saturation or diffusing capacity for carbon monoxide in patients with AFOP, more cases with moderate disease severity were incorporated in our study than previously reported [5]. One reason may be the relatively high percentage of COP which rarely causes marked pulmonary dysfunction. Another might be selection bias; in high-risk patients with severe hypoxia and respiratory failure, we usually do not perform biopsy [43] but rely mainly on serological and radiological findings to initiate treatment as early as possible.

## Conclusions

In summary, several clinical and radiological features were identified in patients with AFOP. It is proposed that AFOP might be an early phase of histologic pattern in the wound healing process after lung injury irrespective of the underlying disease. It may also be a marker indicating intense inflammatory condition with a tendency of recurrence.

## Supporting information

S1 Data (XLSX)
